# Sensitivity of cytology in liver tumor biopsy and its significance in the prompt clinical diagnosis of non‐hepatocellular carcinoma

**DOI:** 10.1002/cam4.5934

**Published:** 2023-04-16

**Authors:** Tasuku Nakabori, Yutaro Abe, Sena Higashi, Kaori Mukai, Azusa Shingetsu, Sanako Nishimura, Sayoko Tsuzaki, Ayumi Ryu, Satoshi Tanada, Shigenori Nagata, Keiichiro Honma, Kazuyoshi Ohkawa

**Affiliations:** ^1^ Department of Hepatobiliary and Pancreatic Oncology Osaka International Cancer Institute Osaka Japan; ^2^ Department of Clinical Laboratory Osaka International Cancer Institute Osaka Japan; ^3^ Department of Diagnostic Pathology and Cytology Osaka International Cancer Institute Osaka Japan

**Keywords:** malignancy detection, minimally invasive technique, rapid on‐site evaluation, tumor histology

## Abstract

**Background:**

Cytology is a fast and simple modality for identifying malignancies and tumor histology. In this study, we analyzed the sensitivity of cytology for liver tumor biopsy and evaluated its potential for prompt clinical diagnosis.

**Methods:**

This retrospective study included patients who had concurrently undergone conventional cytology, on‐site cytology, and histopathology for ultrasound‐guided liver tumor biopsies. In the case of malignant tumors, malignancy was first diagnosed, then preliminary clinical diagnosis was established using histology based on cytology and clinical information, followed by histopathological diagnosis. Sensitivity of malignancy detection was evaluated by comparison with histopathological diagnosis.

**Results:**

Of the 191 tumors, 164 (85.9%) were malignant. The sensitivity of conventional cytology for malignancy detection was 97.6%. The sensitivity of non‐hepatocellular carcinoma (non‐HCC) (99.3%) detection was higher than that of the HCCs (87.5%; *p* = 0.001). The sensitivity of on‐site cytology for malignancy detection was as high as that of conventional cytology. Similar to conventional cytology, the sensitivity of on‐site cytology for non‐HCC detection (99.3%) was higher than that for HCCs (79.2%; *p* < 0.001). In most cases of non‐HCC tumors (126/140, 90.0%), accurate preliminary clinical diagnoses were obtained by combining on‐site cytology with clinical information.

**Conclusion:**

Cytology of liver tumor biopsy has high sensitivity for malignancy, especially in non‐HCC tumors. On‐site cytology can contribute to the prompt clinical diagnosis of non‐HCC tumors when combined with clinical information. This approach may be a reassuring modality for patients with severely advanced cancers requiring prompt clinical diagnosis and quick initiation of treatment owing to their deteriorating health.

## Graphical abstract

Sensitivity of on‐site cytology for malignancy in liver tumor biopsy was comparable to that of conventional cytology. Non‐hepatocellular carcinomas (Non‐HCC) detection sensitivity using conventional (99.3%) and on‐site cytology (99.3%) were significantly high. On‐site cytology may be useful for patients with advanced cancer for quick initiation of treatment, because on‐site cytology with clinical information enabled the clinical diagnosis of most non‐HCC tumors (126/140, 90.0%).

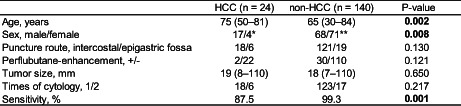

## 1 INTRODUCTION

Two types of tumors develop in the liver, namely, primary lesions and metastatic tumors. Progress in imaging technology has contributed to the accurate diagnosis of liver cancer^1^; however, pathological analysis by liver tumor biopsy is still necessary to confirm the diagnosis, especially in tumors with atypical imaging findings.^2^

Systemic chemotherapy, including immune checkpoint inhibitors, molecular target agents, or cytotoxic agents, has improved survival and disease response in a wide range of malignancies.^3^, ^4^ The progress in treatment options has provided opportunities of good clinical outcomes for patients with advanced cancer as long as the principal organ function is maintained. Patients with advanced cancer can develop multiple organ dysfunction because of metastasis, invasion into multiple organs, malnutrition, or cachexia.^5^ Thus, prompt diagnosis and the quick initiation of anti‐cancer treatment are required in such patients.

Cytology is a simple and minimally invasive technique for the diagnosis of malignancy and histological type. Cytology is utilized to screen for various cancers, such as lung,^6^ uterine cervix,^7^ and bladder cancers.^8^ The on‐site cytology of malignancy at the time of sampling is used for specimen triaging (i.e., rapid on‐site evaluation [ROSE]). The usefulness of ROSE during endoscopic ultrasound‐guided fine‐needle aspiration (EUS‐FNA)^9^, ^10^ and endobronchial ultrasound‐guided transbronchial needle aspiration (EBUS‐TBNA)^11^, ^12^ has been previously reported. However, for liver tumor biopsy, neither the significance nor the clinical usefulness of cytology is clear. In the present study, we analyzed the sensitivity and specificity of cytology for liver tumor biopsy. We also evaluated the contribution of cytology to the clinical diagnosis of malignant liver tumors.

## 2 METHODS

### 2.1 Study population

We retrospectively collected the clinical data of patients who underwent ultrasonography‐guided liver tumor biopsy at Osaka International Cancer Institute, Osaka, Japan, between June 2020 and December 2021. Patients who concurrently underwent conventional cytology, on‐site cytology, and histopathology were included in the analysis. One case in which clinical diagnosis was not possible by liver tumor biopsy was excluded. This study was approved by the Institutional Review Board for Clinical Research at the Osaka International Cancer Institute (approval number: 22056).

### 2.2 Percutaneous liver tumor biopsy

The liver tumor biopsy was performed using a 21‐gauge aspiration needle (Sonopsy‐C1; Hakko). The samples were released from the needle onto the slide using a 20‐mL syringe, and tissue pieces were transferred to a container of 10% formalin for fixation. The slide with the residual specimen was gently imprinted onto another slide. One slide was air‐dried for on‐site cytology, and the other was fixed with 95% ethanol for conventional cytology. The slides for conventional cytology and the 10% formalin container with the tissue specimen were transferred to the diagnostic pathology and cytology department for further analysis. The number of times that on‐site cytology was performed was determined by the operator depending on the result.

### 2.3 Cytology, histopathology, and staining procedure

Conventional cytology was performed by Papanicolaou staining. Histopathological analysis was performed using hematoxylin and eosin (HE) staining, and immunostaining was performed if necessary. These tests were performed in the diagnostic pathology and cytology departments. On‐site cytology was performed at the time of sampling using Shorr staining to reduce analysis time, as previously reported.^13^

### 2.4 Diagnostic process and sensitivity for malignancy

In this study, the diagnosis was made in three steps. First, pathologists reviewed the cytological diagnosis made by cytotechnologists and classified it into positive and negative. If the tumor was diagnosed as malignant, the diagnosis was followed by an evaluation of histological type (i.e., adenocarcinoma, squamous cell carcinoma, or small cell carcinoma). The second was a preliminary clinical diagnosis (such as breast cancer, pancreatic cancer, or biliary tract cancer) by two authors (T.N. and Y.A.), which was completed by combining the histological result of cytology with clinical information of medical history; tumor markers (i.e., the value of carcinoembryonic antigen, carbohydrate antigen [CA19‐9], and alpha‐fetoprotein); and findings of upper or lower gastrointestinal endoscopy, computed tomography (CT), or magnetic resonance imaging (MRI). The third was the final histopathological diagnosis by HE staining or immunostaining of tissue specimens (i.e., well/moderately/poorly differentiated type of hepatocellular carcinoma [HCC] or adenosquamous/mucinous/anaplastic carcinoma of the pancreas). Sensitivity for malignancy was evaluated on the basis of the final histopathological diagnosis.

### 2.5 Statistical analysis

Continuous variables were expressed as the median (range) and compared between groups using the Mann–Whitney *U*‐test. Categorical variables were expressed as numbers and compared between groups using Pearson's chi‐squared test or Fisher's exact test as appropriate. Factors with *p* < 0.10 in the univariate analysis were used in a multivariate logistic regression model. Differences were considered statistically significant at *p* < 0.05. All statistical analyses were performed using SPSS version 20 software (IBM Corp.).

## 3 RESULTS

### 3.1 Sensitivity and specificity of the conventional cytology and analysis of the factor associated with the sensitivity

In this study, 183 patients with 191 tumors were enrolled. The patient characteristics and puncture procedure are shown in Table 1, and the histopathological diagnosis is shown in Table 2. The median tumor size was 21 mm. On‐site cytology was performed once in 164 patients (85.9%), twice in 26 patients (13.6%), and thrice in one patient (0.5%). Among 191 tumors, 164 (85.9%) were malignant and 27 (14.1%) were benign. Among the malignant tumors, pancreatic cancer (60 patients) was the most common, followed by biliary tract cancer (28 patients) and HCC (24 patients). Severe adverse events that required prolonged hospitalization periods did not occur.

**TABLE 1:** Patient characteristics and puncture procedure.

Age, years	66 (30–85)
Sex, male/female	96/87
Puncture route, intercostal/epigastric fossa	160/31
Perflubutane‐enhancement, +/−	43/148
Tumor location, S2/3/4/5/6/7/8	12/14/16/40/41/36/35
Tumor size, mm	21 (7–110)
Times of on‐site cytology, 1/2/3	164/26/1

*Note*: Continuous variables are shown as median (range).

**TABLE 2:** Histopathological diagnosis of the tumors.

Pancreatic cancer	60
Biliary tract cancer	28
Breast cancer	16
Lung cancer	10
NET	9
Colon cancer	5
Pharyngeal cancer	3
NEC	2
Esophagus cancer	1
Bladder cancer	1
Ovary cancer	1
Adrenal cancer	1
Non‐Hodgkin's lymphoma	1
Combined hepatocellular and cholangiocarcinoma	1
MiNEN	1
HCC	24
Inflammatory tumor or abscess	14
FNH	5
Dysplastic nodule	4
Hepatocellular adenoma	1
AML	1
Hemangioma	1
Focal fatty change	1

Abbreviations: AML, angiomyolipoma; FNH, focal nodular hyperplasia; HCC, hepatocellular carcinoma; MiNEN, mixed neuroendocrine‐nonendocrine neoplasm; NET, neuroendocrine tumor; NEC, neuroendocrine carcinoma.

In this study, the first step in the diagnostic process was to confirm if the tumor was malignant or benign by using cytology. Thus, the sensitivity and specificity of conventional cytology for malignancy were determined for all tumors on the basis of histopathological diagnosis. The sensitivity and specificity of the conventional cytology were 97.6% and 100%, respectively. The positive and negative predictive values were 100% and 87.1%, respectively. Factors associated with sensitivity for malignancy were analyzed. The two factors, namely, HCC or non‐HCC (*p* = 0.010), and times of on‐site cytology (*p* = 0.097), which were significant (*p* < 0.10) in the univariate analysis, were included in a multivariate logistic regression model (Table 3). HCC or non‐HCC remained independently associated with sensitivity (*p* = 0.017). Therefore, the patients with liver cancer were divided into HCC and non‐HCC groups. The patient characteristics and puncture procedures in the HCC and non‐HCC groups are shown in Table 4. Patients with non‐HCC were significantly younger than those with HCC (*p* = 0.002). The proportion of males was significantly higher in the HCC group than in the non‐HCC group (*p* = 0.008). There were no significant differences in puncture route, use of perflubutane, tumor size, or times of on‐site cytology between the groups. In terms of sensitivity for malignancy, the non‐HCC group (139/140, 99.3%) was significantly superior to the HCC group (21/24, 87.5%; *p* = 0.001). To analyze the inferiority of the sensitivity for malignancy in the HCC group compared with the non‐HCC group, patients with HCC were classified according to the degree of differentiation (Table S1). Three of the eight well‐differentiated HCCs (37.5%) were negative for malignancy according to cytology, whereas all 14 moderately and two poorly differentiated HCCs were diagnostic for malignancy.

**TABLE 3:** Factors associated with the sensitivity of the cytology.

	Univariate analysis	Multivariate analysis		
	*p*‐Value	OR	95% CI	*p*‐Value
Age	0.191			
Sex	0.637			
HCC or non‐HCC	**0.010**	17.243	1.648–180.392	**0.017**
Puncture route	0.513			
Perflubutane‐enhancement	0.429			
Tumor size	0.261			
Times of on‐site cytology	**0.097**	0.318	0.570–1.770	0.191

*Note*: Bold numbers indicate *p*‐values that meet statistical significance. *p* < 0.1 in the univariate analysis, and *p* < 0.05 in the multivariate analysis.

Abbreviation: CI, confidence interval.

**TABLE 4:** Patient characteristics, puncture procedure, and sensitivity of the conventional cytology in the non‐hepatocellular carcinoma (HCC) and HCC patients.

	HCC (*n* = 24)	non‐HCC (*n* = 140)	*p*‐Value
Age, years	75 (50–81)	65 (30–84)	**0.002**
Sex, male/female	17/4^a^	68/71^b^	**0.008**
Puncture route, intercostal/epigastric fossa	18/6	121/19	0.130
Perflubutane enhancement, +/−	2/22	30/110	0.121
Tumor size, mm	19 (8–110)	18 (7–110)	0.650
Times of cytology, 1/2	18/6	123/17	0.217
Sensitivity, %	87.5	99.3	**0.001**

*Note*: Continuous variables are shown as median (range). Bold numbers indicate *p* < 0.05.

^a^ Three patients had two FNBs for two tumors.

^b^ One patient had two FNBs for two tumors.

### 3.2 High sensitivity and clinical usefulness of the on‐site cytology in patients with liver tumors other than HCC

The sensitivity of on‐site cytology for malignancy was further examined on the basis of histopathological diagnosis. The sensitivities of on‐site cytology for malignancy in the non‐HCC and HCC groups were 99.3% and 79.2%, respectively. The positive and negative predictive values were 100% and 81.8%, respectively. In the non‐HCC group, all results of the on‐site cytology were identical to those of the conventional cytology. By contrast, in the HCC group, two well‐differentiated HCCs were positive (diagnostic) in the conventional cytology but negative (non‐diagnostic) in the on‐site cytology (Table S1). Similar to conventional cytology, on‐site cytology had significantly higher sensitivity for malignancy in the non‐HCC group than in the HCC group (*p* < 0.001). Given that the high sensitivity of on‐site cytology for malignancy was validated in the non‐HCC group, the clinical usefulness of prompt diagnosis by combining the histological results of on‐site cytology with clinical information such as medical history, tumor markers, endoscopy results, and CT or MRI images was further evaluated in the non‐HCC tumor group. Twenty‐six tumors (90.0%) were considered to have a confirmed clinical diagnosis. A representative case is shown in Figure S1. The patient first visited our hospital with hepatic tumors and moderate malaise. Contrast‐enhanced CT showed multiple hepatic tumors with ring enhancement (Figure S1A) and a hypovascular tumor in the pancreatic tail (Figure S1B, arrow). Serological tests revealed elevated levels of liver enzymes, CA19‐9 (>100,000 U/mL; reference range, ≤37 U/mL) and DUPAN‐2 (>4800 U/mL; reference range, ≤150 U/mL). On‐site cytology was positive for malignancy, and the histological diagnosis was adenocarcinoma (Figure S1C). On the basis of these results, pancreatic cancer was preliminarily diagnosed. Subsequently, nab‐paclitaxel plus gemcitabine treatment was initiated as the first‐line chemotherapy for metastatic pancreatic cancer^14^ while the patient was healthy enough to start chemotherapy. A histopathological diagnosis was obtained 3 days later, which was identical to the preliminary clinical diagnosis of pancreatic cancer (Figure S1D).

On the contrary, in 14 tumors, including four poorly differentiated carcinomas (one pancreatic cancer, two intrahepatic cholangiocarcinomas, and one breast cancer); four combined types of carcinoma (two adenosquamous carcinomas of pancreas, one combined hepatocellular and cholangiocarcinoma, and one mixed neuroendocrine‐nonendocrine neoplasm); one neuroendocrine tumor; three tumors (one pancreatic cancer, one colon cancer, and one breast cancer) with the same histological type of multiple primary cancers, which required immunostaining to differentiate; one bladder cancer; and one non‐Hodgkin's lymphoma, the clinical diagnosis remained unclear until the histopathological results were obtained (Table 5).

**TABLE 5:** Diagnosability of the non‐HCC tumors by combining on‐site cytology with clinical information.

	Diagnostic	Non‐diagnostic
	(*n* = 126)	(*n* = 14)
Pancreatic cancer	56	4
Biliary tract cancer	26	2
Breast cancer	14	2
Lung cancer	10	0
NET	8	1
Colon cancer	4	1
Pharyngeal cancer	3	0
NEC	2	0
Esophagus cancer	1	0
Bladder cancer	0	1
Ovary cancer	1	0
Adrenal cancer	1	0
Non‐Hodgkin's lymphoma	0	1
Combined hepatocellular and cholangiocarcinoma	0	1
MiNEN	0	1

Abbreviations: MiNEN, mixed neuroendocrine‐nonendocrine neoplasm; NEC, neuroendocrine carcinoma; NET, neuroendocrine tumor.

## 4 DISCUSSION

Cytology is a widely accepted, simple, inexpensive technique. On‐site cytology is performed at the time of sampling, and it takes only a few minutes (shorter time than conventional cytology). Thus, on‐site cytology is adopted as a specimen triaging method (i.e., ROSE), which is considered useful during EUS‐FNA^9^, ^10^ or EBUS‐TBNA^11^, ^12^ because of the improvement in the diagnostic adequacy rate and reduction in complications and medical costs owing to the decrease in the number of passes per procedure. However, for liver tumor biopsy, the significance and clinical utility of cytology remain unclear. To better understand these problems, the sensitivity of cytology for malignancy and the contribution of on‐site cytology to the prompt clinical diagnosis of malignant liver tumors were investigated in the present study.

The sensitivity of conventional cytology for malignancy according to histopathological diagnosis was 97.6%, which was relatively higher than the 70%–90% in previous reports.^15^, ^16^ The sensitivity of conventional cytology in non‐HCC tumors was significantly higher than that in HCC tumors. The difference arose from the difficulty in diagnosing well‐differentiated HCCs by cytology because three of eight well‐differentiated HCCs could not be diagnosed. The atypia of hepatocytes on cytology is not sufficient to make a definite diagnosis of well‐differentiated HCC because well‐differentiated HCC has little cellular atypia and requires the estimation of structural atypia by histopathology or immunostaining against heat shock protein 70, glutamine synthetase, or glypican‐3.^17^, ^18^

For on‐site cytology, sensitivity for malignancy based on the histopathological diagnosis was as high as that of conventional cytology in both HCC (79.2%, *p* = 0.701) and non‐HCC tumors (99.3%, *p* = 1.000). Similar to conventional cytology, on‐site cytology had higher sensitivity for malignancy in the non‐HCC than in the HCC group (*p* < 0.001). The high sensitivity of on‐site cytology for malignancy in non‐HCC tumors suggested the usefulness of ROSE in liver tumor biopsy as well as during EUS‐FNA^9^, ^10^ or EBUS‐TBNA.^11^, ^12^

One strength of cytology is the quick result within 1–2 days, whereas histopathological analysis requires approximately 5–6 days until the result is obtained. In addition, on‐site cytology can immediately provide results at the same time as sampling. Cytology can provide information on the histological type and malignancy. In this study, the combination of histological diagnosis of on‐site cytology with clinical information enabled us to clinically diagnose most of the non‐HCC tumors (126/140, 90.0%). Some patients with advanced cancer cannot wait to initiate anti‐cancer treatment until the results of the histopathological analysis are received because of deteriorating multiple organ functions. For these patients, cytology may be a reassuring modality because prompt clinical diagnosis made by combining histological results on the basis of on‐site cytology and clinical information could determine the anti‐cancer treatment regimen. Further investigation is required to address this issue. On the contrary, 14 tumors (10.0%), including the poorly differentiated type of carcinoma, combined type of carcinoma, and tumors with the same tissue type of multiple primary cancers that require immunostaining to differentiate, could not be presumed to have a confirmed clinical diagnosis even though histological results by on‐site cytology were combined with clinical information. In these cases, the final histopathological diagnosis by HE staining or immunostaining was followed by the initiation of a suitable anti‐cancer treatment regimen.

There are two major methods of liver tumor biopsy: fine‐needle aspiration biopsy (FNAB) and core needle biopsy (CNB). FNAB is usually performed using a 21‐ or 22‐gauge fine needle under aspiration with a syringe. CNB is usually conducted using a 16‐ to 20‐gauge core needle. These two methods differ in the needle thickness or sampling procedure. It is considered that FNAB using a thinner modality is safer and less invasive but yields smaller tissue. CNB using a thicker modality could provide sufficient material and is preferred for immunostaining to determine tumor origin or ancillary tests to obtain prognostic or therapeutic information.^15^, ^19^, ^20^ In the present study, FNAB using a 21‐gauge aspiration needle was adopted for sampling. We elongated the sampling stroke (approximately 2–3 cm) to obtain enough specimen because the aspiration biopsy needle was thin. Consequently, neither severe procedural complications nor re‐biopsy due to insufficient material occurred in any of the cases. Therefore, FNAB is considered a satisfactory procedure for histopathological examination in most cases.

In this study, patients whose histological diagnosis was made by liver tumor biopsy, were enrolled. One case, which was positive for malignancy in conventional and on‐site cytology but negative for malignancy in histology of biopsy sample, was excluded because histological diagnosis was made by surgical resection. The tumor was 11 mm in diameter. Tumor cells can be efficiently collected by aspiration.^20^ Therefore, in a small tumor, only tumor cells could be collected by an aspiration needle, though sampling of tumor tissue fragments for histology may have been technically difficult.

Our study has several important limitations, including its retrospective nature, single‐center design, and small sample size. On‐site cytology is available at limited institutions because of the heavy burden and considerable efforts on the cytotechnologists and cytopathologists.

In conclusion, the cytology of liver tumors has high sensitivity for malignancy, especially in non‐HCC malignant tumors. Regarding non‐HCC malignant tumors, on‐site cytology could contribute to prompt clinical diagnosis by combining it with clinical information. Cytology, a simple and inexpensive technique, may serve as a safe and prompt diagnostic tool for malignant liver tumors.

## AUTHOR CONTRIBUTIONS

**Tasuku Nakabori:** Conceptualization (lead); data curation (lead); formal analysis (lead); methodology (lead); resources (equal); writing – original draft (lead); writing – review and editing (equal). **Yutaro Abe:** Data curation (equal); formal analysis (equal); resources (equal). **Sena Higashi:** Resources (equal). **Kaori Mukai:** Resources (equal). **Azusa Shingetsu:** Resources (equal). **Sanako Nishimura:** Resources (equal). **Sayoko Tsuzaki:** Resources (equal). **Ayumi Ryu:** Resources (equal). **Satoshi Tanada:** Resources (equal). **Shigenori Nagata:** Methodology (equal); writing – review and editing (equal). **Keiichiro Honma:** Methodology (equal); writing – review and editing (equal). **Kazuyoshi Ohkawa:** Investigation (equal); methodology (equal); writing – review and editing (equal).

## FUNDING INFORMATION

The authors confirm that there are no funding sources to declare.

## CONFLICT OF INTEREST STATEMENT

All authors declare that they have no conflicts of interest.

## APPROVAL OF THE RESEARCH PROTOCOL

The present study was approved by the Institutional Review Board for Clinical Research at Osaka International Cancer Institute (approval number 22056).

## INFORMED CONSENT

Informed consent was obtained, and the opportunity to opt out at any time was provided via a website.

## REGISTRY AND THE REGISTRATION NO. OF THE STUDY/TRIAL

N/A.

## ANIMAL STUDIES

N/A.

## RESEARCH INVOLVING RECOMBINANT DNA

N/A.

## Supporting information


**Figure S1.** A case of pancreatic cancer where the correct clinical diagnosis was made by combining the histological result of on‐site cytology with clinical information. Chemotherapy was initiated before the final histopathological diagnosis was obtained. Contrast‐enhanced CT images show multiple hepatic tumors with ring enhancement (A) and a hypovascular tumor in the pancreatic tail (B, arrow). The on‐site cytology is positive for malignancy, with a diagnosis of adenocarcinoma (C; Shorr staining). The final histopathological diagnosis is adenocarcinoma of the pancreas (D; hematoxylin and eosin staining).

Table S1.

## Data Availability

The data shown in the present study are available from the corresponding author upon reasonable request.
