# Supplementary figures and images for: Sensitivity of cytology in liver tumor biopsy and its significance in the prompt clinical diagnosis of non‐hepatocellular carcinoma
Source: Cancer Med. 2023 Apr 16;12(11):12336–42. doi: 10.1002/cam4.5934 (PMC10278471; doi:10.1002/cam4.5934)

## Slide 1
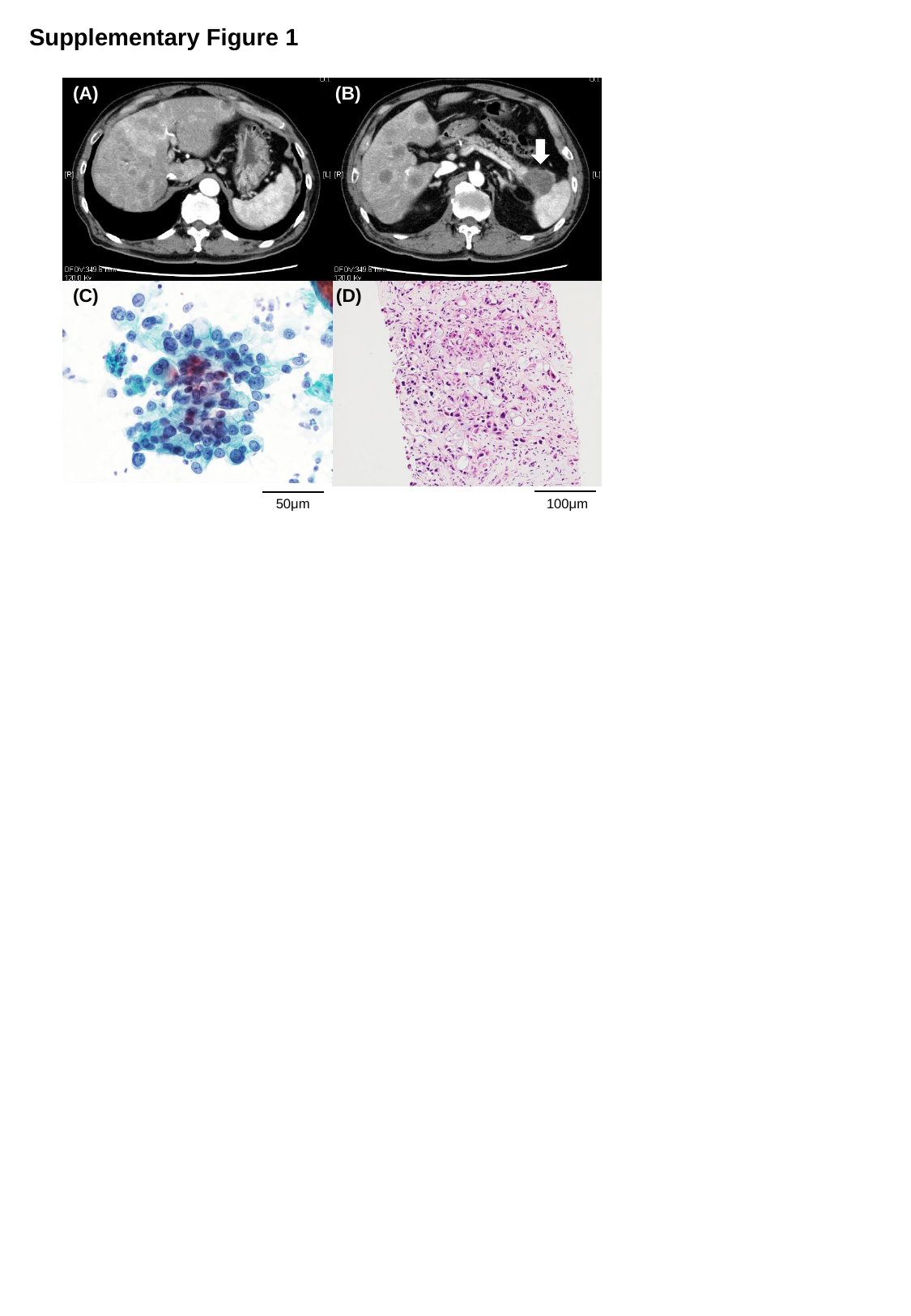

Supplementary Figure 1
(B)
(A)
(C)
(D)
50μm
100μm

Supplement: Supplementary file 1 — Figure S1. A case of pancreatic cancer where the correct clinical diagnosis was made by combining the histological result of on‐site cytology with clinical information. Chemotherapy was initiated before the final histopathological diagnosis was obtained. Contrast‐enhanced CT images show multiple hepatic tumors with ring enhancement (A) and a hypovascular tumor in the pancreatic tail (B, arrow). The on‐site cytology is positive for malignancy, with a diagnosis of adenocarcinoma (C; Shorr staining). The final histopathological diagnosis is adenocarcinoma of the pancreas (D; hematoxylin and eosin staining). [file CAM4-12-12336-s001.pptx]
